# The relationship between glycated hemoglobin A1c levels and exacerbation status in the patients with chronic obstructive pulmonary disease

**DOI:** 10.1186/s13104-022-06217-7

**Published:** 2022-09-22

**Authors:** Behrang Motamed, Ali Alavi Foumani, Azita Tangestaninezhad, Mohammad Almasi, Niloofar Faraji, Alireza Jafarinezhad

**Affiliations:** 1 Inflammatory Lung Diseases Research Center, Department of Internal Medicine, School of Medicine, Razi Hospital, Guilan University of Medical Sciences, Rasht, Iran; 2 Razi Clinical Research Development Unit, Razi Hospital, Guilan University of Medical Sciences, Rasht, Iran

**Keywords:** Glycated hemoglobin A, Pulmonary Disease, Chronic obstructive

## Abstract

**Objective:**

This study was performed in Razi Hospital, Rasht, Iran, between March 2016 and August 2018 on a population of chronic obstructive pulmonary disease (COPD) patients (56 as COPD exacerbation group and 56 as COPD stable group). Study variables include age, sex, occupation, body mass index (BMI), cigarette consumption, duration of COPD, annual hospitalization, dyspnea, glycated hemoglobin (HbA1c), FEV1, and FEV1/FVC indices.

**Result:**

The mean age of the participants was 63.92 ± 10.75 years. There was a significant difference in the hospitalization between the patients with both *exacerbation* and normal state of *COPD* (P ≤ 0.001). HbA1c in the patients with *exacerbation* of *COPD* was significantly higher than stable status (P = 0.001). Logistic regression showed that HbA1c levels and hospitalization were predictors of *exacerbation* of *COPD*. HbA1c levels were statistically significant in terms of hospitalization in patients with COPD exacerbation. There was a significant difference between the HbA1c levels and MMRC in patients with COPD. The percentage of HbA1c was associated with exacerbation of COPD and HbA1c is a good predictor of disease severity in patients with COPD. It also shows that patients with COPD exacerbation and severe COPD are at the higher risk of hyperglycemia.

## Introduction

Chronic obstructive pulmonary disease (COPD) is a Clinical status in which the airways become swollen and narrow and lead to damage to the alveoli. The disease is characterized by airflow restriction [1, 2]. The prevalence of the disease is generally estimated to be 5% [3]. It is estimated that 10% of Iranians have COPD [4]. The main feature of COPD is lung inflammation, which increases with the progression of the disease [5]. Pulmonary comorbidity affects the Prognosis of patients with COPD. Hyperglycemia is seen in 89% of COPD patients during acute respiratory failure who require mechanical ventilation [6, 7]. 50–80% of patients with *exacerbation* of *COPD* suffer from diabetes and are commonly hyperglycemic. This results in longer hospital stays and higher mortality compared to non-diabetic patients [8, 9]. There are various causes of hyperglycemia in COPD patients, including the use of beta-adrenergic drugs and glucocorticoids [10–12]. Other factors such as stress, the pre-exacerbation status of health, systemic features of the disease, inflammatory markers [13, 14], hypoxia [15], and oxidative stress may also be mentioned. Recent studies have shown that HbA1c, which controls basal blood glucose levels, can be a good biomarker for the diagnosis of high-risk diseases [16–20]. In fact, plasma levels of HbA1C in the blood, indicating fasting and postprandial glycemia over a period of 2 to 3 months, are widely used as a long-term marker of glucose [21].

There has been no extensive study on patients with exacerbation of COPD and its consequences in Iran. We hypothesized in this study that HbA1c levels are correlated with COPD severity. As a result, plasma HbA1C may be used as an indicator to predict the clinical status of patients with COPD. The results of this study can also be used to determine the exacerbation status of patients with COPD as a risk factor for pre-diabetes status.

## Patients and methods

### Study design, categorize of patients, and sample size

This cross-sectional study was performed on a population of patients with COPD who referring to Razi Hospital and specialized clinics in Rasht, Iran, between March 2016 and August 2018. Patients with COPD were given the necessary explanations about the purpose of the study and were entered into the study after obtaining informed consent. The diagnosis of COPD in participants was based on the spirometry (JAEGER; Vyntus SPIRO PC spirometer) finding of the “Razi Hospital Respiratory Test Unit” and the Global Initiative for Chronic Obstructive Lung Disease (GOLD) criteria. In this study, modified medical research council (MMRC) questionnaire was used for grading dyspnea. The severity of the disease was determined according to GOLD criteria [20]. Patients were divided into four groups of mild, moderate, severe, and very severe obstruction based on spirometric criteria.

Diagnosis of exacerbation COPD and acute exacerbations of chronic obstructive pulmonary disease (AECOPD) were determined based on the patient’s clinical manifestations, such as aggravated dyspnea, cough, sputum production, and greater use of maintenance treatments, or complementary therapies. In this study, the criteria for COPD exacerbation were dyspnea exacerbation, respiratory distress, hypoxemia (PaO_2_ < 60 mmHg), and hypercapnia (PaO_2_ > 60 mmHg). Interventional or non-intervention mechanical ventilation was required. Staging COPD and the risk of exacerbation were determined according to GOLD criteria. Next, patients were divided into high-risk (C and D) and low-risk (A and B). The normal range for the HbA1C level is between 4% and 5.6%. HbA1C levels between 5.7% and 6.4% show a higher chance of getting diabetes. HbA1C levels of 6.5% or higher also show diabetes. In addition, HbA1C level was measured in Razi Hospital Laboratory, Rasht, Iran, using ELISA method (PishtazTeb, Iran). Sample size was estimated at least 56 participants in each group (95% confidence and 90% test power) based on a study of Haruka et al. [8]. Based on spirometry findings, patients with COPD whose FEV1 / FVC ratio was less than 70%, also with irreversible airway obstruction were included. Patients also with mild, moderate, severe, and very severe obstruction as well as exacerbation COPD were included. In the current study, patients were not included who needed admission to the intensive care unit (ICU). Patients were not included with diabetes, hypoglycemia, diabetic ketoacidosis, hyperglycemic, acute clinical need for intervention and blood transfusion, hemoglobinopathy, Iron deficiency anemia, uremic status, opacity chest X-ray (CXR), and prolonged oral corticosteroid. Patients were excluded who did not wish to enter the study and those who were unable to perform a breath test. Finally, patients were divided into intervention group (Group 1) and control group (Group 2). In the group 1, 56 patients were assigned with COPD exacerbation and AECOPD. In the Group 2, 56 patients were assigned with COPD stable. Spirometry findings and the number of hospitalizations were recorded for the last year of patients with COPD stable.

### Statistical analysis

Variables of the current study Include; age, sex, occupation, body mass index (BMI), cigarette consumption, duration of COPD, annual hospitalization, dyspnea, glycated hemoglobin (HbA1c), FEV1, and FEV1/FVC indices. Mann Whitney and Kruskal Wallis nonparametric tests were used to comparing quantitative variables with abnormal distribution. Chi square tests were used to compare the qualitative variables. Spearman correlation coefficient was used to determine the correlation between variables. Sionel Logistic analyzer was performed to determine the variables related to disease severity. Significance levels of tests were ≤ 0.005 in this study.

## Results

The mean age of the participants was 63.92 ± 10.75 years. According to Table 1, there was a significantly different in the hospitalization between the patients with *exacerbation* of *COPD* and patients with *COPD* in a stable status (P ≤ 0.001) (Table 1). The results of this study showed that HbA1c (5.6%) in the patients with *exacerbation* of *COPD* was significantly higher than in the patients with COPD in the stable status (P = 0.001). Logistic regression showed that HbA1c levels and hospitalization were predictors of *exacerbation* of *COPD* (P ≤ 0.05). Therefore, the exacerbation of COPD was increased to 4.45-fold with an increase in HbA1c levels (P ≤ 0.001). Based on the results, HbA1c levels in terms of severity status of airflow limitation (GOLD) in COPD patients were statistically significant (P = 0.001). According to the GOLD, there was a significant difference between D, A and B groups. As Table 2 shows, there was no significant difference between the HbA1c levels and FEV1 in patients with COPD (P value = 0.076). The study also showed that HbA1c levels were statistically significant in terms of hospitalization in patients with COPD (P = 0.022). Also, there was a significant difference between the HbA1c levels and MMRC in patients with COPD (P = 0.007). There was a significant difference between group 1 and group 3 (Table 2).

**Table 1:** Demographic characteristics of the patients with COPD exacerbation and stable status

Variables					p-value
		**COPD** **stable** **n = 56**	**COPD exacerbation** **n = 56**	**Total** **n = 112**	
Age (Year)		(44.00–87.00) 61.00	(47.00–89.00) 63.00	(44.00–89.00) 61.00	*0.250
Mean (rang)					
Sex	Male	39 (69.6%)	46) 82.1%)	85) 75.9%)	Statistics value 2.39
	Female	30.4) 17%)	17.9) 10%)	24.1) 27%)	**0.122
Cigarette consumption	No	25) 44.6%)	20 (35.7%)	45) 40.2%)	Statistics value 0.93
	Yes	31) 55.4%)	36) 64.3%)	67) 59.8%)	**0.335
Underlying disease	No	28) 50.0%)	26) 46.4%)	54) 48.2%)	Statistics value 0.14
	Yes	28) 50.0%)	30) 53.6%)	51.8) 58%)	**0.705
During of disease (Year) Mean (range)		(1.00–25.00) 5.00	(2.00–30.00) 8.00	(1.00–30.00) 5.00	*0.003
High blood pressure	No	38 )67.9% (	31 )55.4% (	69 )61.6% (	Statistics value 1.85
	Yes	18 (32.1%)	25 (44.6%)	43 (38.4%)	0.174**
Cardiovascular disease	No	39 (69.6%)	43 (76.8%)	82 (73.2%)	Statistics value 0.73
	Yes	17 (30.4%)	13 (23.2%)	30 (26.8%)	0.393**
Frequency of hospitalization	None	25 (44.6%)	6 (10.7%)	31 (27.7%)	Statistics value 19.06
	Once	19 (33.9%)	30 (53.6%)	49 (43.8%)	> 0.001**
	Twice	6 (10.7%)	16 (28.6%)	22 (19.6%)	-
	Three times and more	6 (10.7%)	4 (7.1%)	10 (8.9%)	-
Frequency of exacerbation	None	8 (14.3%)	2 (3.6%)	10 (8.9%)	Statistics value 20.08
	Once	26 (46.4%)	9 (16.1%)	35 (31.3%)	> 0.001**
	Twice	10 (17.9%)	24 (42.9%)	34 (30.4%)	-
	Three times and more	12 (21.4%)	21 (37.5%)	33 (29.5%)	-

*Mann Whitney Test ** Chi Square Test

**Table 2:** HbA1c status in patients with COPD base on the intensity of air flow restriction in the GOLD; FEV1, MMRC (n = 112)

	Categories	Number of patients	HbA1c Mean (rang)	p-value
The intensity of air flow restriction in the GOLD	A	6	^a^5.05 (4.90-6.00)*	0.001**
	B	18	^a^5.25 (4.90-7.00)*	
	C	6	^ab^5.35 (5.10–6.20)*	
	D	82	^b^5.96 (4.90–8.10)*	
Severity of COPD base on FEV1	Mild	9	*(6.20–4.90) 5.10	0.076**
	Moderate	60	*(7.20–4.90) 5.90	
	Severe	35	*(8.10–4.90) 5.90	
	Very severe	8	*(7.20–5.20) 5.90	
Frequency of exacerbation (Year)	None	31	*(00.7–90.4) 50.5 ^a^	0.022**
	Once	49	*(10.8–90.4) 10.6 ^b^	
	Twice and more	32	*(20.7–90.4) 85.5 ^ab^	
The Modified Medical Research Council (MMRC)	1	10	*(20.6–90.4)10.5^a^	0.007**
	2	35	(20.7–90.4) 80.5 ^ab^	
	3	47	(10.8–90.4) 10.6 ^b^	
	4	20	(10.7–90.4) 75.5 ^ab^	

*Identical lowercase letters indicate no statistically significant difference in the Mann-Whitney comparative test with Bonferroni correction (p > 0.017)

**Kruskal Wallis Test

Glycated hemoglobin (HbA1c); Global Initiative for Chronic Obstructive Lung Disease (GOLD); forced expiratory volume in one second (FEV1), Modified Medical Research Council (MMRC) (n = 112)

According to the results of the study, there was a weak significant correlation between HbA1c levels and MMRC in patients with COPD (Spearman’s Correlation Coefficient, r = 0.197, P = 0.19). Also, there was no significant correlation between HbA1c levels and FEV1, FEV1 / FVC and duration of disease in patients with exacerbation of COPD. Also, HbA1c levels were not significantly correlated with FEV1, FEV1 / FVC and disease duration in patients with COPD in the stable status (Table 3).

**Table 3:** Correlation of glycosylated hemoglobin (HbA1c) with the forced expiratory volume in one second (FEV1), FEV1/ forced vital capacity (FVC) and duration of disease in patients with COPD exacerbation, and stable status

	Variables	HbA1c	
COPD exacerbation	FEV1%	Spearman correlation coefficient	0.106
		P value	0.438
		Type of correlation	No significant correlation
	FEV1/FVC	Spearman correlation coefficient	0.199
		P value	0.141
		Type of correlation	No significant correlation
	Duration of disease (Year)	Spearman correlation coefficient	0.047
		P value	0.732
		Type of correlation	No significant correlation
COPD exacerbation	FEV1%	Spearman correlation coefficient	0.254-
		P value	0.580
		Type of correlation	No significant correlation
	FEV1/FVC	Spearman correlation coefficient	0.097
		P value	0.475
		Type of correlation	No significant correlation
	Duration of disease (Year)	Spearman correlation coefficient	0.118
		P value	0.386
		Type of correlation	No significant correlation

## Discussion

Chronic obstructive pulmonary disease is one of the leading causes of mortality worldwide and its prevalence is likely to increase in the next twenty years [22]. Glucose metabolism disorders are more prevalent among COPD patients. Recent evidence has shown that diabetes can lead to COPD prognosis [23]. Recent studies have shown that high HbA1c levels, which control basal blood glucose levels, are a good biomarker for the diagnosis of serious diseases [16, 20]. Plasma HbA1C levels, which indicate fasting and postprandial glycemia over 2 to 3 months, have been widely used as a long-term marker of glucose regulation [24]. In this study, the frequency of hospitalization in patients with *exacerbation* of *COPD* was more than that of the Stable group, and this difference was statistically significant (P ≤ 0.001) (Table 1). Also, the frequency of exacerbation (twice a year or more) was higher in patients with *exacerbation* of *COPD* than in patients with COPD in stable status (P ≤ 0.05). In this study, HbA1c levels were significantly higher in patients with *exacerbation* of *COPD* in terms of GOLD.

In the present study, the percentage of glycosylated hemoglobin (HbA1c) by disease severity based on FEV1 levels in COPD patients was not statistically significant (P = 0.076). In the Stojkovikj et al., study [25], the prevalence of diabetes mellitus in all COPD patients was severe and very severe, 21%. In this study, in the group with very severe COPD, the median HbA1c was significantly higher than in the group with severe COPD. This is consistent with our results. Yang’s study [26] also showed that changes in HbA1c levels were significantly correlated with COPD severity. Other studies have also shown that patients with diabetes, FVC, FEV1, maximal expiratory flow (PEF), and reduced vital capacity (VC) had more obstructive or deleterious patterns than other patients [27, 28]. The study showed a U-shaped relationship between HbA1c and the incidence of COPD [29]. It has been shown that the risk of COPD is higher among people who have HbA1c levels below 6% or greater than 10% than those with HbA1c levels between 6% and 7%. Similar to the results in this study, found no difference in the prevalence of diabetes in patients with PFEV1 compared with patients who did not find PFEV1 [30]. However, a similar study by Baba et al. [31] showed that PFEV1 / FVC was associated with HbA1c. According to the Baba study patients with high glucose (100 ≤ FPG mg / dl and 5.6% HbA1c) had the lowest FEV1: FVC ratio [25]. Specifically, individuals with an FEV1/FVC index are significantly higher than HbA1c. This difference may be due to differences in host populations as well as the difference in severity of COPD in the population. In addition, non-diabetics may have a history of diabetes. Because consuming glucose-lowering drugs can hurt performance, research has shown that patients with type 2 diabetes who have HbA1c levels above 10% or below 6% are at a significantly increased risk of COPD [29]. Researchers have found that corticosteroid-consuming patients with acute respiratory failure (ARF) have significantly lower HbA1c levels and a longer hospital stay than patients who do not take corticosteroids [26]. According to our results, the percentage of HbA1c is positively correlated with the severity of COPD, which also increases with the increase in HbA1c percentage of COPD. Therefore, it seems that HbA1c can be used to predict the status of COPD patients and vice versa, using COPD severity as an indicator to predict pre-diabetic conditions. Despite these results, the impact of HbA1c percentages on pulmonary function parameters (and vice versa) is not well understood.

The strength of this study was evaluating dyspnea grade and severity of the disease by MMRC and GOLD criteria, respectively. Furthermore, future studies are expected to better investigate the prevalence of diabetes in the COPD population, its association with pulmonary parameters, and the association with the prognosis of patients with COPD without a history of diabetes.

## Conclusion

Our findings represented that the percentage of HbA1c was associated with exacerbation of COPD and that HbA1c was a good predictor of disease severity in patients with COPD. It also shows that in our study patients with exacerbated COPD and those with severe involvement‌ are more at risk for hyperglycemia.

## Limitations

In this study, healthy subjects were not included in the control group to more accurately examine the variables and indices. Also, another limitation of this study were the limited sample size, some incomplete patients’ clinical data, and lack of follow-up of patients.

## Data Availability

The datasets used and/or analyzed during the current study are available from the corresponding author on reasonable request.

## Abbreviations

COPD: Chronic obstructive pulmonary disease
GOLD: Global Initiative for Chronic Obstructive Lung Disease
MMRC: Modified Medical Research Council
AECOPD: Acute Exacerbations of Chronic Obstructive Pulmonary Disease
ICU: Intensive care unit
